# Editorial: Children's Exercise Physiology, Volume II

**DOI:** 10.3389/fphys.2022.864661

**Published:** 2022-03-07

**Authors:** Ana Filipa Silva, Luca Paolo Ardigò, Luis Paulo Rodrigues, Hermundur Sigmundsson, Matthieu E. M. Lenoir, Wook Song, Filipe Manuel Clemente

**Affiliations:** ^1^Escola Superior Desporto e Lazer, Instituto Politécnico de Viana Do Castelo, Rua Escola Industrial e Comercial de Nun'Álvares, Viana Do Castelo, Portugal; ^2^The Research Centre in Sports Sciences, Health Sciences and Human Development (CIDESD), Vila Real, Portugal; ^3^Research Center in Sports Performance, Recreation, Innovation and Technology (SPRINT), Melgaço, Portugal; ^4^Department of Neurosciences, Biomedicine and Movement Sciences, School of Exercise and Sport Science, University of Verona, Verona, Italy; ^5^Norwegian University of Science and Technology, Trondheim, Norway; ^6^Reykjavik University, Reykjavik, Iceland; ^7^Department of Movement and Sports Sciences, Ghent University, Ghent, Belgium; ^8^Institute of Sport Science, Seoul National University, Seoul, South Korea; ^9^Institute on Aging, Seoul National University, Seoul, South Korea; ^10^Instituto de Telecomunicações, Delegação da Covilhã, Lisboa, Portugal

**Keywords:** children, physical exercise, physical fitness, motor development, youth sport

After the previous highly successful Frontiers Research Topic “Children's Exercise Physiology” (2020), we have challenged the academic community to continue exhibiting their main findings in volume II of this topic. In the past edition, the main topics of research were focused on performance, physical fitness, measurement methodologies, motor competence, overweight and obesity, and pathological subjects. In the current volume II, the Research Topics were less dispersed focusing on two main domains: (i) sports training; and (ii) exercise for health.

In the sports training, there was a specific dedication to characterize the physiological demands imposed by the training process in youth soccer players, while the other studies tested relationships between maturation and selection for competition and identified adjusted loads to exercise intervention on the patellar tendon.

Regarding the domain of exercise for health, there was a focus on understanding the interactions between exercise and maturation and executive functions in young, while one study focused on understanding the exercise role on fat oxidation. Within this domain, a validation of a motor competence assessment tool was also introduced.

Considering the relevance of the topic, we invite our colleagues to follow the read the summary of the evidence presented in our Frontiers Research Topic.

## Sports Training

Two studies (Nobari, Ruivo Alves, et al.; Nobari, Vahabidelshad, et al.) centered the research into monitoring the psychophysiological demands (measured by the rate of perceived exertion) of under-16 soccer players during the season. One of the studies (Nobari, Ruivo Alves, et al.) has tried to establish the hypothesis of a dose-response relationship between accumulated training demands and the variations in physical fitness. Although different outcomes have been considered (e.g., maximal oxygen uptake, heart rate, linear sprint, locomotor response in an intermittent progressive multistage test), only maximal oxygen uptake variations were partially explained by accumulated training demands. Interestingly, peak height velocity also partially and significantly explained variations of aerobic power.

Also using data from the monitoring process in under-16 players, Nobari, Vahabidelshad, et al. analyzed variations of training demands in different micro and mesocycles. The rate of perceived exertion using the CR-10 Borg's scale was used as an outcome for calculating the weekly acute and chronic load. The within-week analysis revealed that training sessions 3 days before the match are the ones with similar demands to the match, while a decrease of intensity and volume is observed in the 2 days closer to the match. Considering the comparisons between moments of the season, it was found that the middle season was the most demanding.

With the aim to analyze the influence of anthropometry, body composition, and maturation on accumulated time of play and matches as a starter in young soccer players, seventy-nine youth soccer players from under-11 to under-14 were analyzed for 6 months (Clemente et al.). Results showed that no significant differences in accumulated minutes of play and matches as a starter were found between the two levels of maturation assessed. Also, maturity offset, leg length, and sitting height were largely correlated with minutes of play. However, the models to explain the variation of minutes of play and matches as a starter only explained 35 and 12%, respectively, with the lean mass, leg length, and sitting height as the greatest contributors. Therefore, this study suggested that maturation is not necessarily a determining factor in the participation of matches since anthropometry and body composition was shown to play an important role. However, the authors also highlighted that those results should be interpreted with caution due to the context of this study design.

Mersmann et al. investigated in male handball players (age 12–14 years) the effects of an over-season functional high-load training designed to facilitate tendon adaptation and reduce muscle-tendon imbalances on patellar tendon pain occurrence, knee extensor strength, and architecture in addition to patellar tendon mechanical properties. Two groups (control, following a common strength training plan; and experimental, with the functional high-load training for the patellar tendon in addition) were assessed at four time points. From about half competition period until season end 30% of controls reported patellar tendon pain occurrence or worsening, whereas the experimental group remained pain-free. Knee extensor strength and vastus lateralis thickness increased similarly in both groups without any tendon stiffness or maximum tendon strain changes. Both groups showed the same tendon strain over-season pattern. Overall, assessed functional high-load training decreases patellar tendon pain occurrence in adolescent athletes despite no tendon strain decrease.

## Exercise For Health

Two studies in our Frontiers Research Topic centered the research on the executive functions of young populations (Cabral et al.; Laureys et al.). One of the studies (Cabral et al.) tested a hypothesis of funding relationships between cardiorespiratory fitness and performance in executive functions of male and female adolescents. Executive functions included the test of planning and problem-solving abilities, cognitive flexibility, inhibitory control, working memory, and sustained attention, while cardiorespiratory fitness was defined as the performance obtained in a progressive multi-stage test. Interestingly, results found in the research revealed that those with better cardiorespiratory fitness were the same with better planning and problem-solving abilities and cognitive flexibility.

In a different approach, Laureys et al. tested the effect of chronological age, maturation on executive functions of both male and female adolescents. Interestingly, inhibition, working memory, planning, and shifting were positively associated with age and maturation in boys. On the other hand, in girls, maturation only significantly affected inhibition. This means that age and maturation are associated with executive functions, although with a different magnitude of relationships when considering sex.

The substrate utilization during submaximal exercise, plus the glycemic responses and hormonal counter-regulation to exercise were analyzed in a population of 24 pre-pubescent children (50% of them with type 1 diabetes mellitus—T1DM) (Fel et al.). The entire sample completed a submaximal incremental exercise test to determine their fat and carbohydrate oxidation rates by indirect calorimetry. It was also monitored the levels of glycemia, glucagon, cortisol, growth hormone, noradrenaline, adrenaline, and insulin were monitored until 120 min post-exercise. When comparing children with and without T1DM, a significantly lower absolute peak oxygen uptake (VO2 peak), lower absolute maximal lipid oxidation rate, and lower exercise power in children with T1DM was observed. The overall carbohydrate and lipid oxidation rates were the same in the two groups, however, in intensity exercises higher than 50% of VO2 peak, fat oxidation rate was significantly lower in the pre-pubescent children with T1DM. Finally, the change in glucose levels in response to exercise was negatively correlated with the pre-exercise glucose concentration. In conclusion, this study suggested that T1DM-related dysregulation in the metabolic and hormonal response to exercise even in pre-pubescent children with T1DM who have adequate glycemic control.

Motor competence is a critical key point for an active and healthy life. Aiming to provide a tool to the academic and practical community, Coppens et al. aimed to validate a compact form of Körperkoordinationstest für Kinder (KTK3). The study conducted on 2,248 children revealed that the inclusion of only four tests was powerful enough to differentiate sexes, ages, and sports participation. The article also provided normative values to those who want to compare values.

### Looking to the Future

Although many developments in children's exercise physiology some topics still need more research. For example, the identification of intra-individual variations regarding training interventions and specific consideration about dose-response relationships are topics that should be more investigated. Moreover, testing the effectiveness of minimal-effective doses in comparison to regular exercise doses should be also analyzed aiming to identify how to implement exercise-based programs in short breaks at school or at home. Additionally, more explanation about physiological mechanisms underlying physical adaptations and responses to training stimulus should be conducted. These are only a few examples of how more research in children's exercise is needed. Thus, we motivate our colleagues and ourselves to continue researching children's exercise physiology aiming to provide a more solid body of knowledge and also help the community to be more effective in the practical scenarios.

## Author Contributions

AS, LPA, and FC written, revised, and approved the article. LR, HS, ML, and WS approved the article. All authors contributed to the article and approved the submitted version.

## Conflict of Interest

The authors declare that the research was conducted in the absence of any commercial or financial relationships that could be construed as a potential conflict of interest.

## Publisher's Note

All claims expressed in this article are solely those of the authors and do not necessarily represent those of their affiliated organizations, or those of the publisher, the editors and the reviewers. Any product that may be evaluated in this article, or claim that may be made by its manufacturer, is not guaranteed or endorsed by the publisher.

